# Advances in Smart Sensing and Medical Electronics by Self-Powered Sensors Based on Triboelectric Nanogenerators

**DOI:** 10.3390/mi12060698

**Published:** 2021-06-15

**Authors:** Min Jiang, Yi Lu, Zhiyuan Zhu, Wenzhu Jia

**Affiliations:** 1 Faculty of Mathematics and Information Science, Southwest University, Chongqing 400700, China; jm0506@email.swu.edu.cn; 2 School of Optoelectronic Engineering, Chongqing University of Posts and Telecommunications, Chongqing 400065, China; luyileo@cqupt.edu.cn; 3 Faculty of Engineering, Zhejiang University, Hangzhou 316021, China

**Keywords:** TENG, self-powered sensors

## Abstract

With the rapid progress of artificial intelligence, humans are moving toward the era of the intelligent connection of all things. Therefore, the demand for sensors is drastically increasing with developing intelligent social applications. Traditional sensors must be triggered by an external power source and the energy consumption is high for equipment that is widely distributed and working intermittently, which is not conducive to developing sustainable green and healthy applications. However, self-powered sensors based on triboelectric nanogenerators (TENG) can autonomously harvest energy from the surrounding environment and convert this energy into electrical energy for storage. Sensors can also be self-powered without an external power supply, which is vital for smart cities, smart homes, smart transportation, environmental monitoring, wearable devices, and bio-medicine. This review mainly summarizes the working mechanism of TENG and the research progress of self-powered sensors based on TENG about the Internet of Things (IoT), robotics, human–computer interaction, and intelligent medical fields in recent years.

## 1. Introduction

In 2012, Wang et al. [1] proposed the triboelectric nanogenerator (TENG), and since then, an increasing number of research studies have been conducted in this field [2,3,4,5,6]. Recently, Wang et al. [7] re-defined the TENG and elaborated on the different concepts of triboelectric electrification (TE) and contact electrification (CE). As a result, TENGs have been widely applied to different research fields [8,9], such as wind energy, water energy [10], flexible electronics, and self-powered sensors [11], owing to their unique advantages of low-frequency, irregular, and distributed energy harvesting [12,13,14].

Self-powered sensors and systems are constructed of a compos capable of sensing, communication, controlling, and responding. However, energy harvesting and storage are also crucial for the system [11]. In general, self-powered devices can be realized by harvesting solar energy, electromagnetic energy, mechanical energy, and other external energy sources. In addition, a self-powered sensor [13] can act as a sensor that can automatically send out electrical signals without an external power supply. Further, it can provide self-generated power as working power for the sensor when activated and in sleep mode. Additionally, it can realize autonomous operation without any external battery to provide energy and solve the problem of the power supply and energy consumption of sensors. Therefore, self-powered sensors represent a significant direction in the sensor network. Based on the above advantages, Wang et al. have conducted much research on self-powered sensors since 2005. As a result, they have been successfully applied in many fields, including wearable devices, smart sports, and smart transportation [15].

**Figure 1:**
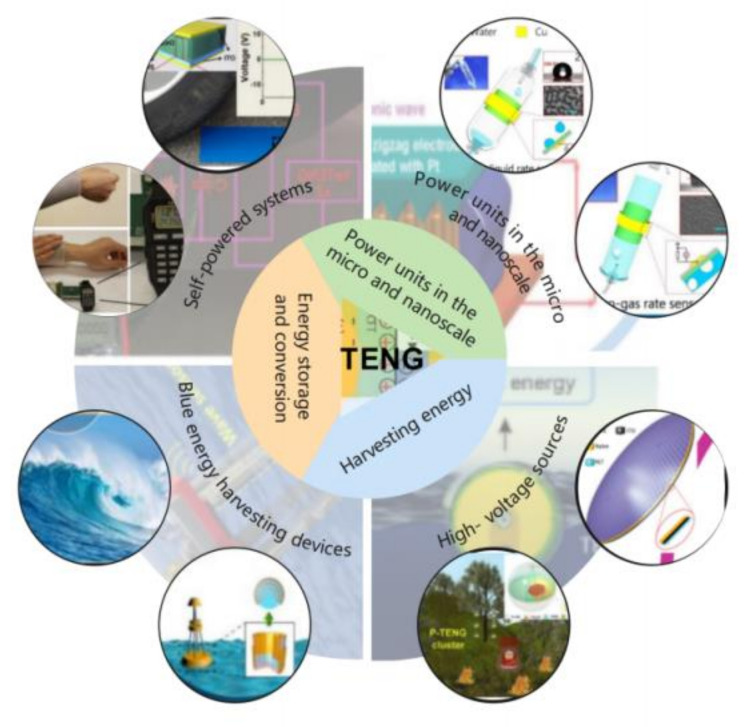
Schematic illustration of the application of TENGs.

The self-powered sensor based on TENG uses Maxwell’s displacement current [16] as the driving force to efficiently convert mechanical energy into electrical energy. Therefore, it can autonomously trigger electrical signals without an external power source [17,18] to become self-powered [19,20]. Additionally, the sensor has the characteristics of a flexible and straightforward structure [21,22,23], a high level of integration, and low cost [24,25,26,27,28,29,30,31]. Based on these unique advantages of TENG, the sensor has broad application potential (Figure 1) in the fields of micro/nano energy [32,33,34,35,36,37], self-powered sensing [38,39,40,41,42], high-voltage power supply, [43] and blue energy [44,45,46,47,48,49], and can play a crucial part in solving the problems of energy collection, signal collection, power supply, and energy conversion [50,51,52,53,54,55,56,57].

Medical health has always been the focus for researchers. However, for the health and medical equipment, problems such as large power consumption, excessive structure size, and the inconvenience of operation are common [58,59,60,61,62,63,64]. Therefore, there is an urgent demand to develop compatible and sustainable medical equipment for healthcare. The self-powered sensor based on TENG realizes the possibility of self-powering, which can transform mechanical energy into electricity by the coupling effect of triboelectrification and electrostatic induction [65]. In addition, it also has the advantages of broad material selectivity and high-charge density, which can solve the above problems effectively. Therefore, researchers have carried out extensive research on TENG in the medical field [66,67,68,69,70,71]. At present, there are three main applications of TENG in the field of smart medical treatment. First, as a sensor, it can output electrical signals and collect information for human stimulation. Second, TENG is utilized to generate electrical stimulation to achieve medical treatment directly. Third, medical devices based on TENG can realize the real-time monitoring of human physiological signals, implant organs to treat diseases, and integrate systems for remote monitoring.

**Figure 2:**
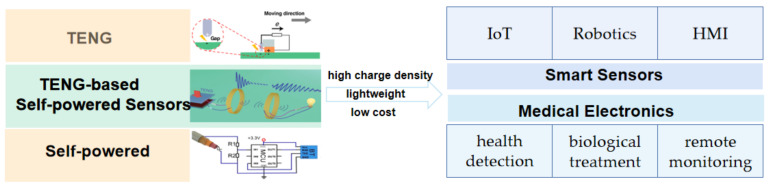
Schematic illustration of TENG-based Self-powered Sensors.

This review will introduce the latest progress of TENG-based self-powered sensors in smart sensors and medical applications (Figure 2), TENG’s working mechanism, and the basic principles of self-powered sensors.

## 2. Working Principle of Triboelectric Nanogenerators

### 2.1. First Principle Theory of TENG

Wang invented TENG in 2012 [72] by using the coupling effect of contact CE and electrostatic induction to convert mechanical energy into electrical energy/electrical signals effectively. Notably, TENG is not limited to nanomaterials. Instead, the driving force of TENG is Maxwell’s (Formula 1) displacement current, which is the current generated by the changing electric field plus the polarization change rate of the medium [73].

(1) $$\nabla \times H=J\prime +\frac{\partial D\prime }{\partial t}$$

(2) $$J_{D}=\frac{\partial D}{\partial t}=\varepsilon \frac{\partial E}{\partial t}+\frac{\partial P_{S}}{\partial t}$$

In 2019, Wang extended the expression of the displacement current [74] and first introduced the Ps term in the current displacement vector D to derive the output power of TENG as the first principle of the theory for quantifying the output and electromagnetic behavior of the nanogenerator (Formula 2). The Ps term is the polarization density caused by the surface static surface electricity generated by mechanical triggering and the dielectric polarization P caused by different electricity and electric fields. Thus, regardless of whether there is an external electric field, this surface electrostatic charge can generate piezoelectric polarization and triboelectricity.

### 2.2. CE and TE Mechanisms

When two materials come into contact without rubbing against each other, CE occurs. However, TE involves the friction between two materials, which is typically inseparable from TE. Therefore, CE is a physical concept in science, while TE is a practical engineering approach for situations wherein friction and friction debris may exist [74]. Moreover, CE is a common physical phenomenon in solid–solid, solid–liquid, liquid–liquid, gas–gas, and gas–solid interactions. The electrical mechanism between solid and solid [75] is similar to that under external pressure: the electron clouds of two atoms overlap, which reduces the potential barrier between the two atoms, and thus the electrons can jump between atoms. The CE from solid–liquid [76] is caused by the formation of an electric double layer. Wang’s group proposed that the electrical double layer (EDL) formation be divided into two steps. The first step is transferring the electrons when the solid surface is in contact with the liquid surface. The second step is reacting ions between the solid and the liquid surface, which results in the gradient distribution of anions and cations at the solid–liquid interface. Experiments have confirmed the existence of both electron transfer and ion transfer during the solid–liquid electrification process. This discovery has had a significant impact in the fields of interface chemistry and electrochemistry. Therefore, the two-step model is also called the Wang model for EDL.

### 2.3. Triboelectric Nanogenerators

The working modes of TENG are the contact-separation mode, lateral sliding mode, single-electrode mode, and free-standing mode [77]. When two polymer films are in contact and separate from each other for the contact-separation mode, the tribe-charges resulting from CE cause a potential difference in the interface area and the back electrode, which leads to a current flow if an external load connection exists. In the lateral sliding mode, when two dielectric films are in contact, frictional charges are generated between the two materials, polarization is formed in the horizontal direction, and electrons are driven to flow between the two poles and generate electric current. In single-electrode mode, when the size of TENG is limited, the upper charged object approaches or leaves the lower object, which changes the local electric field distribution, and a current is formed from the electron exchange between the lower electrode and the ground. Finally, in free-standing mode, the back of the dielectric layer is plated with two unconnected symmetrical electrodes, and the reciprocating movement of a charged object between the two electrodes changes the potential difference between the two electrodes, which drives electrons back and forth between the two electrodes through an external circuit load flow.

## 3. Self-Powered Sensors

### 3.1. Self-Powered Theory

The triboelectric nanogenerator has intrinsic capacitance characteristics. Its equivalent circuit is a voltage source and inherent capacitance in series, equivalent to an AC power supply. Therefore, TENG is required to be integrated to form a self-powered system, which stores the generated charge and outputs it in a prescribed manner in the commercial electronic equipment area. [78]

In 2006 [79], Wang proposed a self-powered system, which stores the output of the nanogenerator in a capacitor and periodically sending it to a sensor to measure the blood glucose or blood pressure. The self-powered concept [80] combines energy harvesting modules, circuit management modules, and energy storage modules to form a self-powered energy unit that can continuously supply energy to other devices by harvesting energy from the environment.

### 3.2. The Development of Self-Powered Sensors

In electrochemistry, researchers collect environmental energy to achieve self-powered pollution degradation [81], hydrolysis [82], anti-corrosion, and air pollution filtration [83], as well as self-powered electrochromic devices for smart window systems [84], which can be combined with electrochemical workstation-driven electrochromic devices. The comparison of color-causing devices shows the substantial progress of self-powered systems [11]. In the human–computer interaction, the researchers extended the basic principle of self-powered pressure sensing to the keyboard structure and developed a pressure/touch sensor to realize the self-powered human–computer interaction function [85,86]. Researchers also realize the application of self-powered biomedical sensors to detect sound waves, heartbeats, and cardiovascular detection by analyzing the corresponding electrochemical signals generated by the excitation in the environment [87,88,89,90,91,92,93].

Self-powered sensors can effectively harvest and convert external energy into electrical energy/electrical signals based on their advantages and use in a wide range of applications in various fields. Table 1 shows the comparison of a different type of self-powered sensors.

## 4. Self-Powered Sensors Based on Triboelectric Nanogenerators

### 4.1. Application of TENG-Based Self-Powered Sensors in IoT

**Figure 3:**
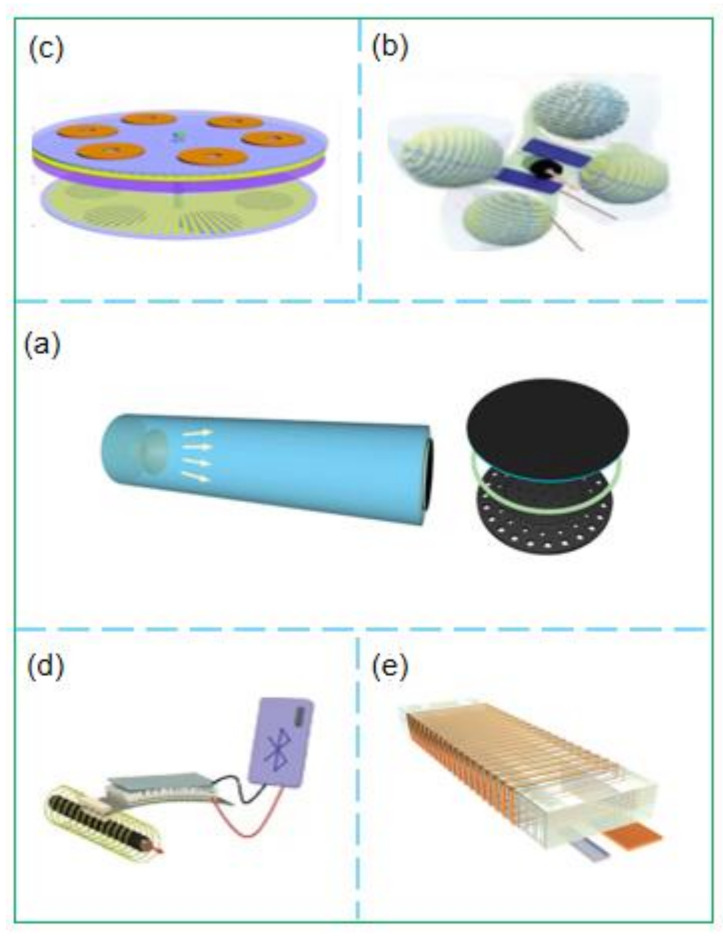
(**a**) Schematic illustration of the A-TENG device [95]; (**b**) Schematic illustration of the AoPS [96]; (**c**) Schematic illustration of the designed hybridized nanogenerator [97]; (**d**) Schematic illustration of the indoor wireless positioning system [99]; (**e**) Schematic illustration of the design and working mechanism of MDC-TENG [100]. (**a**) Reprinted with permission, LN: 5067400553203. (**b**) Reprinted with permission, LN: 5067401239029. (**c**) Reprinted with permission, LN: 5067401479131. (**d**) Copyright 2021, RSC Publishing.

The IoT is an indispensable setting for the development of the digital age. Studies have reported that there will be more than 30 billion devices connected to the IoT by 2025. A traditional sensor node consumes 4 mW of energy. If traditional sensors are used to construct the IoT, 120 million kilowatts will be required, equivalent to burning about 15 tons of standard coal per hour [94]. As self-powered sensors based on TENG have the characteristics of low energy consumption, self-power, and high energy collection efficiency, they can effectively overcome the energy consumption problem. The study of Yuan et al. [95] found that a 3D-printed acoustic friction nanometer self-powered edge sensing system (Figure 3a), which comprises a generator and control circuit, can harvest sound energy and convert voice signals into electrical signals. Experimental results have revealed that the system can generate 4.33 mW of output power under 100 dB  of sound pressure level excitation. This discovery expands the application potential in the field of low-power and low-cost intelligent IoT. However, it is challenging for IoT systems in unstable and harsh environments to use widely distributed electronic devices for energy harvesting fully. Xu et al. [96] developed a hybrid all-in-one self-powered sensor (Figure 3b) by mixing a high-performance TENG with solar cells to harvest various types of energy from the environment. Through calculation and analysis using the finite element method, it was concluded that four TENG units could be used to obtain almost continuous direct current and achieve a high average power of 5.63  mW, which can power 1160 LED lights simultaneously and can also power electronic equipment under all weather conditions. This equipment improves the utilization rate of energy and lays a reliable foundation for developing IoT applications. To maximize the conversion of the collected energy into electric energy and effectively transmit it, Chen et al. [97] developed a rotating, disc-type electromagnetic triboelectric hybrid generator based on the TENG self-powered sensor (Figure 3c). Experimental results reveal that 130 mA of current and 217.8 mW of electrical energy can be output under a 20 k ohm load and stored in a supercapacitor. The wireless transmission of electric energy through a pair of commercial spiral coils is sufficient for charging a mobile phone at a distance of 100 cm. This discovery can be effectively applied to energy collection and wireless transmission in IoT systems. Chen et al. achieved high-efficiency output energy and energy utilization by using an energy management solution that converts the low-frequency energy collected by TENG into high-frequency energy by using the adjustable opening voltage of the Spark switch [98]. They found, through experiments, that under a 2.4 mm air gap, TENG can provide up to 7.5 kv to turn on the Spark switch, which is much higher than the traditional energy management work. The successful application of the universal, efficient, stable, and low-cost energy management solution reported in this work on the triboelectric nanogenerator shows the vast potential of this energy management solution in the distributed energy supply of the IoT. Lim and Zhao have also made particular contributions to the investigation of efficient energy harvesting. Lim et al. [99] introduced a modified nanostructure to design a high-performance magneto-mechano-triboelectric generator (MMTEG) self-powered sensor (Figure 3d), which can convert magnetic energy into electric energy. Through the alternating magnetic field of 7 oe, the open-circuit voltage generated by MMTEG can reach 708  V. To optimize the triboelectricity, and electrostatic breakdown of the direct-current friction nanogenerator, Zhao et al. [100] introduced the structural factor  K, which is related to the electrode structure, and adjusted K by adjusting the electrode structure and size to increase the charge density. The experimental results revealed that the charge density increased with the size of the device (Figure 3e). This study provides a new solution for large-scale energy harvesting in the IoT. Owing to its unique structure, MMTEG can be combined with micro-nano processing technology to manufacture fine-structured devices, providing a strategy for the miniaturization of IoT energy sources.

**Figure 4:**
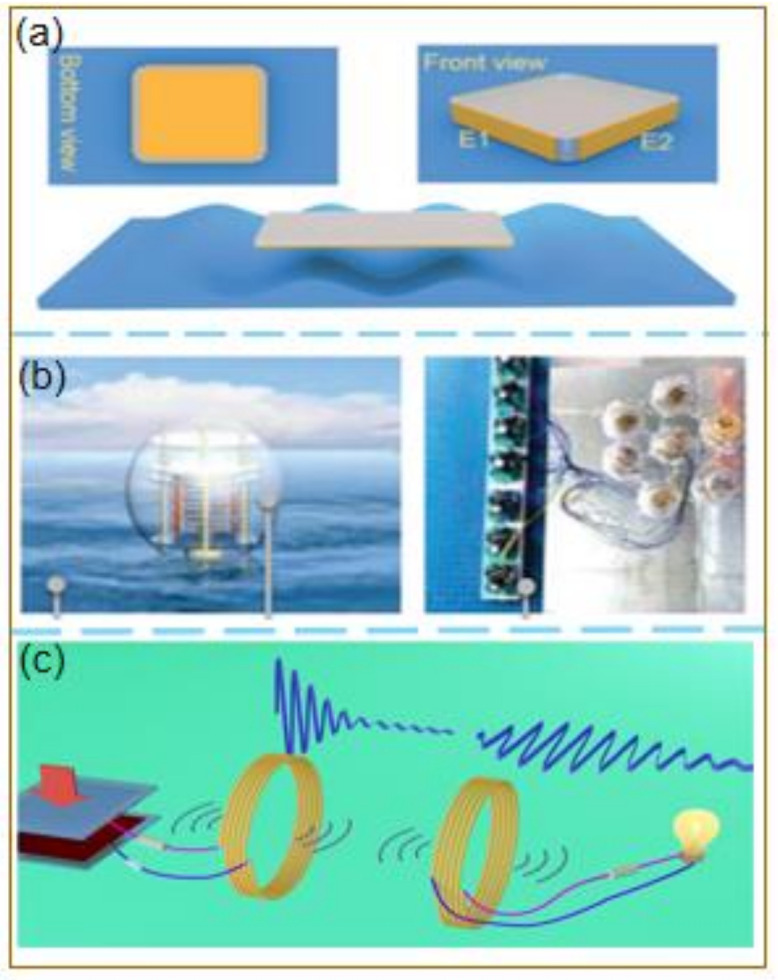
(**a**) (I): Schematic illustration of self-powered motion vector sensor based on DC-TENG, (II): Schematic illustration of the conventional self-powered sensors based on AC-TENG [101]; (**b**) Schematic illustration of a single spherical TENG with spring-assisted multilayered structure floating on the ocean surface [103]; (**c**) Magnetic resonance coupled wireless TENG [106]. (**a**) Reprinted with permission, LN: 5067420482614. (**b**) Reprinted with permission, LN: 5070090891241.

Signal acquisition is another important factor determining the development of an IoT system. The study of Yin et al. [101] on signal collection considered TENG-based self-powered sensors and found that TENG-based friction vector sensors (TVS) (Figure 4a) can measure various motion parameters while being self-powered. These parameters are free of interference from environmental electromagnetic signals, and the sensitivity is greatly improved. This study introduced new ideas for the design of motion vector sensors to collect information in the IoT. Moreover, research on self-powered sensors based on TENG has also made breakthrough progress in maritime IoT. Ding et al. developed a new low-cost, hand-driven water purification device: TriboPump [102]. The device is based on a coaxial mechanical structure that integrates a special disc-shaped friction nanogenerator (D-TENG) and a tubular electric field-assisted copper ion sterilizer (CECIC), which can effectively collect the mechanical energy that drives the mechanical water pump while pumping water. Liang et al. [103] developed a new type of charge excitation circuit (CEC) with the advantages of high integration, high efficiency, and minimum impedance. The self-powered sensor integrated with the TENG network (Figure 4b) can achieve 208 times the enhanced current of 25.1 mA. Additionally, it can transmit radio frequency (RF) signals for remote environmental monitoring and power wireless communication systems. This study provides a strategy for improving the transmission performance of the TENG network in water wave energy harvesting and provides new guidance for building a maritime IoT system.

Regardless of whether the IoT is industrial or maritime, the electricity demand will continue to grow, and the conversion of mechanical energy into electrical energy has become a feasible approach for satisfying this demand. Liu et al. [104] developed a sliding-type DC-TENG and rotating-type DC-TENG, which can realize constant current output by coupling the frictional electrification effect and electrostatic breakdown, respectively. Thus, the energy storage unit can be charged without using a rectifier. In 2017, Chen et al. [105] developed a practical bionic-jellyfish triboelectric nanogenerator (bjTENG) that used a polymeric thin film as the triboelectric material. The structure can reach a sustainable output performance of 143  V, 11.8 mAm^−2^, and 22.1 μCm^−2^ under a low frequency of 0.75 Hz and a 60 cm depth of water. These electrical energies can be directly used to drive dozens of LEDs or temperature sensors. Then, Zhang et al. [106] investigated a wireless “green” power supply (Figure 4c). By integrating micro-switches and self-powered sensors based on friction nanogenerators, the pulse voltage output is converted into a sinusoidal voltage signal with a fixed frequency, which plays a role in self-power. This approach provides an essential direction for green sustainable energy to realize the development of the IoT.

Compared with battery-powered sensors, self-powered sensors based on TENG can be used as IoT sensor nodes. The sensors can harvest energy from the environment to provide electrical energy, collect environmental signals, and contribute to green and sustainable development. However, based on existing research, there are still many problems and challenges inhibiting the commercialization of these sensors, such as the material selection, manufacturing process, packaging technology, product durability, stability, functionality, and sensor stability.

### 4.2. Application of TENG-based Self-Powered Sensors in Robotics Field

**Figure 5:**
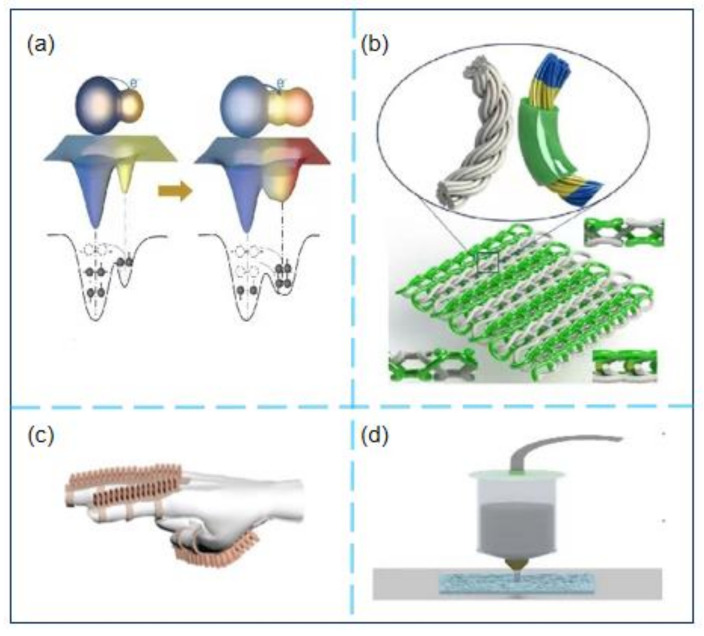
(**a**) Schematic illustration of an electron-cloud-potential-well model proposed to explain the charge transfer between spider silk and PET with/without PVDF (arrows indicate electron transfer events) [108]; (**b**) Schematic illustration of the fabrication process of the core-sheath yarn and 3DFIF-TENG[111]; (**c**) Schematic illustration of the assisting glove[114]; (**d**) Schematic illustration of the 4D Printed TENG[115]. (**a**) Reprinted with permission, LN: 5067420482614. (**b**) Reprinted with permission, LN: 5070100466333. (**c**) Reprinted with permission, LN: 5067550253136. (**d**) Reprinted with permission, LN: 5067410691194.

The harvesting of distributed, green, and sustainable energy from the environment [107] to realize smart machines is an important direction for future scientific and technological development. Self-powered sensors based on TENG can efficiently harvest and use universal low-frequency mechanical energy from the environment, contributing to the solution of the energy crisis problem. Huang et al. [108] developed a “self-matching” friction-piezoelectric coincidence nanomachine (Figure 5a) with significantly improved energy output. They used a gas-phase-induced phase separation process and a matching method of structure and surface potential, and their device can be used to harvest energy for robots. The self-healing elastomer developed by Xiong et al. [109] enables the gas–solid interaction friction nanogenerator GS-TENG to collect mechanical energy during the deformation process and plays a vital role in collecting energy for soft robots. Yi et al. [110] also investigated deformable and retractable self-powered sensors and included a conductive liquid in a polymer cover. The shape-adaptive triboelectric nanogenerator (saTENG) unit can effectively collect energy in various working modes. Through experiments, it was found that saTENG can reach 300, which effectively solves the deformable power supply problem of robots. The research groups of Chen et al. [111] and Zou et al. [112] expanded the application fields of self-powered sensors based on TENG by improving the material genome and double-needle flat knitting machine technology (Figure 5b). Wu et al. proposed a cylindrical self-powered multifunctional sensor (MS) with a translational rotary magnetic mechanism based on the TENG [113]. Experiments showed that the output performance of the friction nanogenerator is not only related to the friction material, but can also be enhanced by using an electrode material with a small work function, low resistance, and suitable surface topography. In addition, the sensor can detect faint stimuli of 0.01 ms^−2^ and as small as 0.8 N. The research has provided a good application prospect in motion monitoring, safety protection, and robotics.

In the field of robotics, power supply and drive problems have constantly challenged researchers. Chen et al. [114] designed a high-sensitivity triboelectric self-powered angle sensor (SPAS) (Figure 5c) and an inductive pneumatic soft actuator (PSA) by combining TENG in the cavity. Their experimental results reveal that the self-powered sensor can feed back the dynamic and static state of PSA, and the output of TENG has a great linear relationship with the bending angle. This study introduced new ideas for achieving the precise control of robots. Later, Huang et al. [115] reported that, by introducing 4D printing technology to make a transparent self-healing TENG (Figure 5d), the maximum output power density can reach 56 mWm^−2^ when collecting mechanical energy, which can be used for monitoring. The self-powered sensor of the bending angle of the human body joint is used to sense and control the precision structure of the robot. The TENG-based self-powered sensors make a big difference in the robot’s energy harvesting and power supply equipment. Guo et al. reported [93] a self-powered triboelectric auditory sensor (TAS) using TENG technology. Compared with traditional piezoelectric cochlear implants, TENG technology has medium- and low-frequency broadband response characteristics, higher signal output intensity, a single channel, and cheap preparation. The research results express the huge application prospects in solving the challenges of next-generation intelligent robots. However, the size of the devices used in the robotics field has exceptionally high limits. To simplify the manufacture of self-powered sensors, Wu [116] and others investigated a mechanoreceptor based on potential-triboelectric single-mode drive, which can significantly simplify the operation of the device and measuring circuit. This self-adaptive self-powered induction mechanism can detect and recognize static and dynamic mechanical stimuli and thus has enormous potential for use in intelligent robot systems.

Although TENG cannot yet replace industrial power plants and provide electricity to thousands of households, the self-powered sensor based on TENG can play a crucial role in low-frequency and discrete sensors owing to its unique advantages. Furthermore, in terms of energy collection and conversion, the efficiency of TENG is greatly improved compared with traditional generators, and TENG is extensively used in the field of robotics.

### 4.3. Application of TENG-based Self-Powered Sensors in Field of Human–machine Interfaces

**Figure 6:**
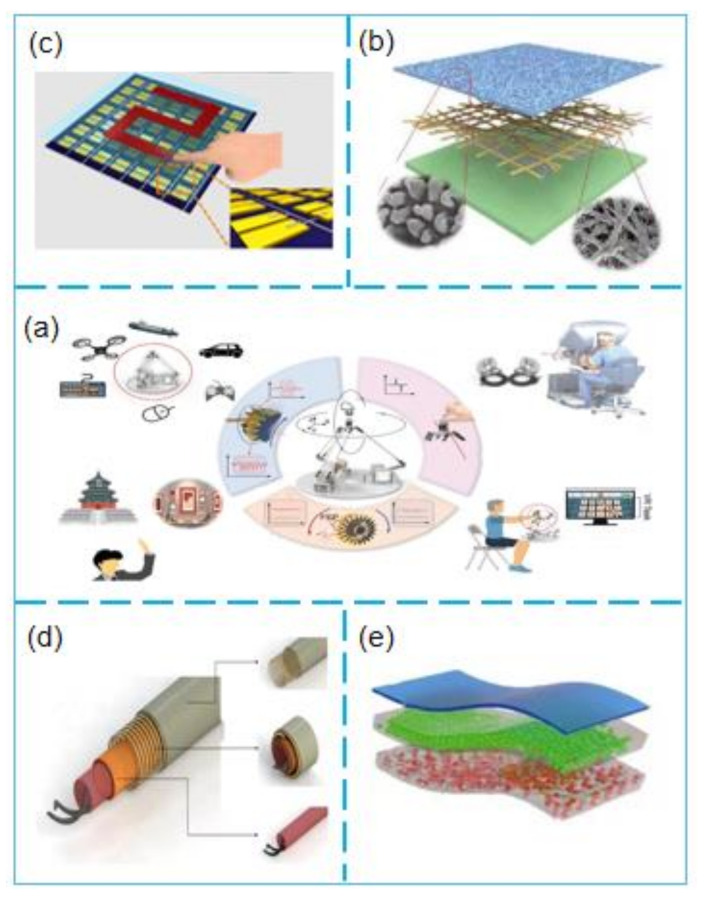
(**a**) Schematic illustration of the DT-HMI for diversified applications[117]; (**b**) Schematic illustration of the single-electrode ultrathin stretchable SI-TENG [118]; (**c**) Schematic illustration of the proposed touchpad showing the drawn pattern of number [119]; (**d**) Schematic illustration of the energy fiber for energy harvesting, storage, and utilization (including TENG, sensor, and SC) [120]; (**e**) Schematic illustration of the device. Insets: Surface and cross-sectional SEM images of the electrification layer (FEP) with surface modification [121]. (**a**) Copyright 2021, John Wiley & Sons. (**b**) Reprinted with permission, LN: 5070121150675. (**c**) Reprinted with permission, LN:5067410340372. (**d**) Reprinted with permission, LN: 5070100466333. (**e**) Copyright 2021, American Chemical Society.

In recent years, with the continuous development of smart sensing, human–computer interaction technology has gradually entered a period of rapid development on a global scale and is gradually expanding into various fields. Hou et al. [117] developed self-driven delta-parallel human–machine interface (DT-HMI) technology for 3D sensing and control (Figure 6a). They used three pairs of TENG induction gears to obtain information on the positive and negative rotations and rotation angles of the gears through contact separation and sliding modes and thereby calculated the spatial position and movement posture of the DT-HMI. Their research results can be effectively applied to self-powered interfaces to realize the remote monitoring and mapping of spatial locations. Jiang et al. [118] extensively investigated the human–machine interface power supply. They fabricated a skin-inspired triboelectric nanogenerator (SI-TENG) through the synchronous electrospinning of TPU and pointed spraying of AgNWs (Figure 6b). Through experiments, they found that the open-circuit voltage of the generator is 95 V. Additionally, the power density can reach 6 mWm^−2^, and SI-TENG has excellent energy harvesting and self-powered induction capabilities. This discovery has a wide range of applications in the field of human–machine interfaces. However, the traditional human–machine interface has strict power requirements and has problems such as having a complex structure. Yun et al. [119] used a 49-pixel TENG array on a flexible substrate to form a self-powered triboelectricity-based touchpad (TTP) (Figure 6c) working in touch and sliding mode. The classification accuracy can reach up to 93.6% when the neural network is pre-trained, and the touchpad has excellent compatibility and can become a functional human–machine interface. As TENG can effectively harvest low-frequency and irregular mechanical energy, Han [120] and others combined the fibrous TENG, energy storage unit, and sensor into the same unit to create a multifunctional coaxial energy fiber (Figure 6d). Their experimental results revealed that the maximum output power density of energy harvesting in single electrode mode is 2.5 µW. Therefore, this is a novel approach for energy harvesting in human–machine interfaces.

To solve the wireless communication problem of human–computer interaction, Su et al. [121] investigated a new type of self-powered visualized flexible pressure sensor (SP-VFPS) based on a micro-compression-porous structure and single-electrode friction nanogenerator matrix-integrated self-powered sensor system (Figure 6e). The system can respond to the tribe-electroluminescence (TIEL) generated by the vertical pressure in real-time through experiments. The device has high sensitivity (S > 190 kPa^−1^) in a wide pressure range and has a fast response time of less than 10 ms, which is a good solution for the wireless optical communication of robot applications in human–machine interfaces.

### 4.4. Application of TENG-based Self-powered Sensors in Field of Smart Medicine

**Figure 7:**
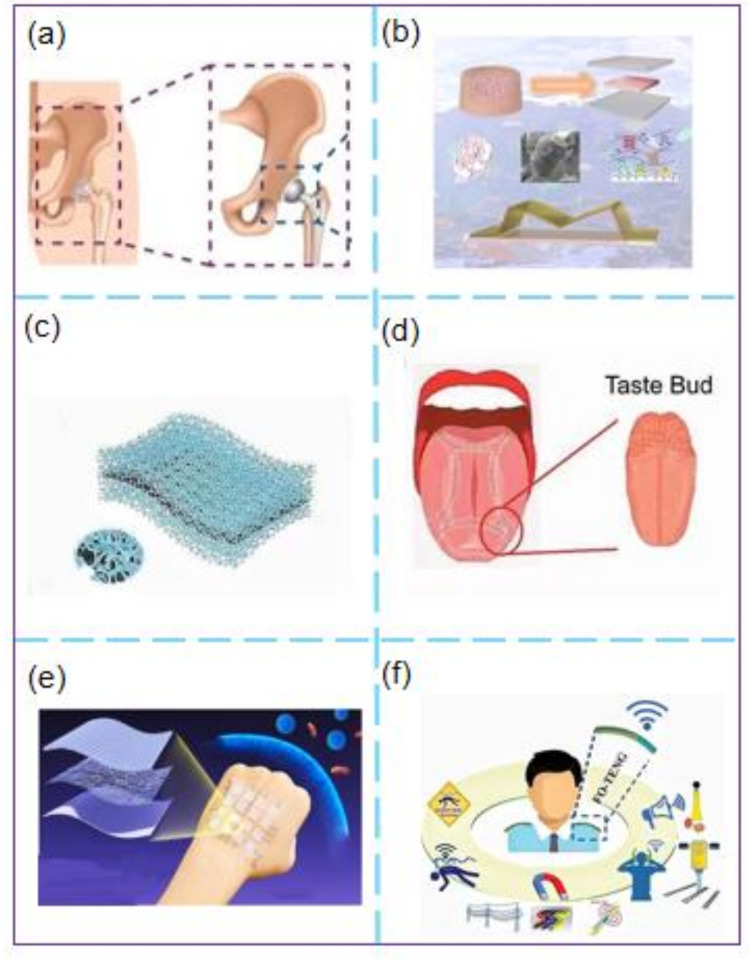
(**a**) Schematic illustration of the wear debris sensor [122]; (**b**) Stretchable and Flexible tremor sensor [123]; (**c**) Schematic illustration of the T-Foam [124]; (**d**) Schematic illustration of the taste receptor: taste bud [127]; (**e**) Schematic illustration of a transparent and antibacterial electronic skin is designed for sensitive tactile sensing [129]; (**f**) Schematic illustration of the hazard preventive wearable platform [133]. (**a**) Reprinted with permission, LN: 5067420140637. (**b**) Reprinted with permission, LN: 5070121150675. (**c**) Copyright 2021, John Wiley & Sons. (**e**) Reprinted with permission, LN: 5067551067416. (**f**) Reprinted with permission, LN:5070130451862.

With the development of smart sensors, artificial joints have gradually become a standard medical method. Liu et al. [122] developed a self-powered sensor based on TENG (Figure 7a) and used it to compare the self-powered sensor in a joint wear simulator. Their experimental results revealed that the sensor could be used to realize the real-time monitoring of wear debris. This study is significant for the development of smart medicine.

As an essential branch of intelligent healthcare, health monitoring has been increasingly attracting attention. However, most currently developed wearable devices require an external power supply, which is unsuitable for sensing low-frequency body movements. Kim et al. [123] synthesized a catechol-chitosan-diatom hydrogel triboelectric nanogenerator (CCDHG-TENG) (Figure 7b) using marine biological materials. The open-circuit voltage is 110 V, the short circuit current is 3.8 μA, and the peak power density is 29.8 mWm^−2^. The CCDHG-TENG can be used to collect energy generated by human movement and can also be used with self-powered tremor sensors to identify the health status of Parkinson’s patients through machine learning algorithms. Wu et al. [124] proposed a triboelectric foam (T-Foam) self-powered sensor comprising foam material and embedded electrodes (Figure 7c). In this case, a PTFE plate is used to drive the experiment. The output power of the sensor is up to 5.46 mW, and the sensor can be integrated into insoles and other products to develop smart applications for health monitoring, which provides a new approach toward the development of smart medicine. Wang et al. studied a nanogenerator based on Kapton film and PET film on the bottom as an effective three-electric layer [125]. Experiments showed that short-circuit transfer charge (QSC) and open-circuit voltage are linearly related to displacement and bending angle. In addition, KT-TENG is connected to human knee joints and fingers and can detect and monitor knee joints and gestures, demonstrating that it has numerous potential applications in the fields of real-time human motion monitoring.

In recent years, using TENG-based self-powered sensors to prepare electronic devices for biomedical applications has always been a critical research direction for researchers, and significant progress has been made. Wang’s research team proposed to use TENG as a motion sensor and energy harvester [126], which can convert biomechanical motion into electrical energy so that ion infiltration can be carried out without the need to store energy, so hydrogel-based ones have side-by-side electrodes. As a result, the soft patch can realize non-invasive ion penetration into transdermal drug delivery (TDD). Furthermore, this self-powered wearable ion conductance TDD system can be driven and adjusted by the energy obtained by biomechanical motion for closed-loop motion detection and treatment. Zhao et al. [127] developed a self-powered flexible acid taste sensor based on frictional electrification/enzyme-reaction coupling (Figure 7d) to detect ascorbic acid concentration. This sensor can collect energy from human movement and thus eliminates the need for an external power supply. Additionally, the sensor can be used to construct an electronic tongue for taste receptors. Zhang et al. developed a breath-driven single-electrode triboelectric nanogenerator (TENG) [128], which can absorb mechanical energy from the airflow of human breathing and generate corresponding electrical signals to deliver control command through breathing for the HMI interaction. The research can reduce the threshold of using modern electrical equipment and personal electronics for disabled people. Zhu et al. [129] used the self-powered characteristics of TENG to prepare a sensor layer with uniform and well-defined nanostructures (Figure 7e), which can be effectively attached to any curved surface to achieve the function of tactile perception. It provides new guidance to the selection of electronic equipment for carrying out intelligent medical treatment. Among them, the pacemaker is a successful example of a TENG-based self-powered sensor in smart medical applications. In 2014, Wang’s research team [130] used TENG to harvest energy from breathing and convert it into electrical energy. The voltage of 3.73 V AC was output to power a prototype pacemaker. This research and development effort demonstrated that TENG-based self-powered sensors could be applied to pacemakers for the first time. Two years later, the same group improved TENG and achieved a TENG self-powered pacemaker [131] into a large animal. In 2019, they developed [132] a novel fully self-powered “symbiotic cardiac pacemaker,” and the energy obtained from the body of a large animal was used to achieve a complete pacing function. Thus, a significant breakthrough was realized in the research and development of self-powered CIED and provided good proof-of-concept for applying electronic equipment to biological therapy. Additionally, TENG-based self-powered sensors have made great strides in the application of remote medical monitoring. For example, Hassan [133] et al. reported a multi-modal ferrofluid-based triboelectric nanogenerator (FO-TENG) based on magnet fluid (Figure 7f). The self-powered sensor mainly comprises a deformable elastomer tube filled with magnetic fluid and surrounded by copper wires. Through experiments, it was found that FO-TENG is stretchable, highly flexible, and can be used in telemedicine. Thus, the application problem of intelligent multi-faceted sensing platforms is effectively solved by monitoring.

The self-powered sensor based on TENG can collect the energy in the environment, store and convert it into electrical energy. Compared with traditional power supply equipment, the self-powered sensor significantly reduces energy consumption. Therefore, the sustainable and green sensor has been successfully applied in many fields. Table 2 shows the application of some sensors based on TENG in different fields.

Based on currently available technology, there are still many challenges inhibiting the application of TENG-based self-powered sensors in health care, such as the stability, biocompatibility, and biosafety of electronic devices when they are in close contact with the human body. Secondly, concerning pacemakers, the area of the electronic equipment will interfere with the contraction of the heart; therefore, it is necessary to use a novel structure and ultra-thin packaging materials to prepare electronic devices. Furthermore, related issues such as power management, wireless transmission, and evaluation standards must also be resolved. However, TENG-based self-powered sensors are very suitable for applications in the healthcare and medical fields. The developed self-powered electronic medical equipment has high integration capability, extended working time, high sensing capability, real-time data transmission capability, and other functions that fit the human body’s needs.

## 5. Summaries and Perspectives

This paper reviewed the progress of research on TENG-based self-powered sensors in smart sensors and medical electronics, including IoT, robotics, human–machine interface, and smart medical electronic devices applications. The self-powered sensor based on TENG has the advantages of small size, wide material selection, and simple preparation. Moreover, it can convert and store electrical energy harvested from the surrounding environment. Therefore, this sensor has a more environmentally efficient and adequate power supply function.

Although significant research progress has been carried about TENG-based self-powered sensors in recent years, there are still many inadequacies inhibiting their commercial availability. This paper discussed problems on energy harvesting, structure miniaturization of smart sensors, and smart medical electronic device applications in the medical field. In addition, this review also discusses the challenges faced by the application of TENG-based self-powered sensors in the field of smart sensing and medical electronics as follows.

### 5.1. Smart Sensing

*Miniaturization*: With advanced nanofabrication and microfabrication techniques [22], smart sensors can become increasingly compact and integrated. Being self-powered based on TENG technologies that harvest energy from the surrounding environment might mean that smart sensors no longer need to be powered by external batteries. To miniaturize self-powered smart sensors, while maintaining output power, several possible research directions should be considered, such as designing novel structures for higher outputs, using ultrathin encapsulation materials and methods, and applying flexible integrated electronic circuits.

*Output performance*: The development of TENG-based self-powered sensors as smart sensing devices with high output performance is a continuous challenge. According to in-depth theoretical research on TENG [74], searching for advanced triboelectric materials and the structure design of TENGs is essential. Some possible approaches can be considered, such as modifying the existing structure to improve the output performance of devices. Recently, introducing the structure factor K in the electrode structure has been demonstrated as an effective method to increase the output charge density and improve performance [99]. In addition, adding a liquid lubricant between the friction materials has proven to be an effective way to reduce materials abrasion [115]. Therefore, using the solid–liquid friction materials instead of rigid materials can reduce abrasion to improve output performance.

### 5.2. Medical Electronics

*Interfacing with the human body*: As smart sensors are used in medical systems, the device must contact the human body in a close and stable manner. However, given the curvilinear and soft features of human organs that the sensor will be implanted into, the inability of rigid wearable materials and implantable systems to form a tight bond remains difficult. Two methods can be considered to prepare self-powered devices that solve the above problems. First, develop more advanced materials, such as flexible materials and fiber materials. Second, further optimization of the structural design of smart sensors should be pursued, which might include the incorporation of ultrathin, porous, and woven to introduce flexibility and stretchability for the entire sensor.

*Wireless communication technology*: Since the existing wireless communication mechanism requires a large amount of power consumption [93], it is necessary to distribute low-energy and low-cost sensors to collect and transmit data. However, as the distance and frequency of wireless transmission continue to increase, the requirements for communication functions have become more stringent. Therefore, by optimizing wireless technologies such as internal circuits, transmitters, and communication networks and developing self-powered energy can enable TENG-based self-powered sensors to be applied in medical electronics fields extensively.

## Figures and Tables

**Table 1:** Comparison of various self-powered sensors.

Self-Powered Sensor	Materials	Performance	Application
Water Splitting Sensor [82]	Kapton/FR4/Au/Cu	transformed efficiency (77.9%)	tribo electrolysis
Active Sensors [86]	PTFE/Nylon/PET/ITO	superior sensitivity (51 mVPa^−1^)	wearable medical/health monitoring
Pressure Sensor [87]	PDMS/Cu/PTFE	high mechanical durability, excellent robustness behavior, high elastic property	human electronics interaction
Gesture Sensor [88]	Nylon/PDMS/PTFE/Cu	high sensitivity (0.77 VkPa^−1^), ultrawide range of pressure detection (from 0.2 kPa to 500 kPa)	gesture monitoring, sign language interpretation system, human–machine interface application
Liquid/Gas Sensor [89]	PE/PTFE	smaller capillary gets a higher sensitivity	micro total analysis system
Heart Sensor [90]	PDMS&Parylene/PTFE/Ti/Kapton/Au/Spacer/n-PTFE/Al	monitoring heart rates’ accuracy (99%)	healthcare industry
Keystroke Sensor [91]	Cu/Al/PTFE	keystroke identification accuracy (99%)	authentication system
Tactile Sensor [92]	human skin/PDMS	sensitivity of the pressure (0.29 ± 0.02 VkPa^−1^)	machine interfacing, micro/nano-electromechanical systems, touch pad technology
Auditory Sensor [93]	Kapton/Au/FEP	ultrahigh sensitivity (from 100 to 5000 Hz,110 mVdB^−1^)	biomedical sensor, intelligent healthcare

**Table 2:** Comparison of various self-powered sensors based on TENG.

Materials	Device Area (mm^2^)	Voc (V)	Isc (uA)	Performance	Application
Cu/Nylon/FEP [95]	78.5	5240	3.76	lighted 1160 LEDs	IoTs, implantable medical devices
AgNWs/TPU [118]	4 × 10^4^	95	0.3	high stretchability (~800%)	human–machine interface, security systems
Nylon/PDMS [120]	7.065	10	0.25	high sensitivity (1.003 V·kPa^−1^)	human−machine interactive system, intelligent robotic skin, security tactile switches
PU Foam [124]	4.55625 × 10^5^	2300	15	lighted 186 light-emitting diodes	motion, health, safety monitoring
